# Geo-hazards assessment and land suitability estimation for spatial planning using multi-criteria analysis

**DOI:** 10.1016/j.heliyon.2023.e18159

**Published:** 2023-07-10

**Authors:** Oana-Elena Chelariu, Ionuț Minea, Corneliu Iațu

**Affiliations:** Alexandru Ioan Cuza University of Iași, Faculty of Geography and Geology, Department of Geography, Iași, Romania

**Keywords:** Multi-hazard, Suitability map, Spatial multi-criteria analysis, Geospatial techniques, Moldova catchment

## Abstract

The fundamentals contribution of multi-hazard assessment lies in its ability to guide and identify directions for territorial development, aiming to reduce vulnerability through the implementation of appropriate measures. In the present study, Multi-Criteria Decision Making supported by Geographic Information System was utilized to solve spatial problems related to territorial sprawl. Considering this perspective, an analysis was conducted on the susceptibility of land to the occurrence of geo hazards in the Moldova catchment, situated in the northeastern region of Romania, within a transitional zone between mountains and plateaus. The analysis focused on assessing the likelihood of geo-hazards like floods, landslides, and earthquakes, utilizing the weights obtained through the implementation of the Analytical Hierarchy Process (AHP). Subsequently, the obtained results were utilized to generate a multi-hazard map, which facilitated the identification of areas that are suitable for territorial development. The results were validated in two ways: by sensitivity analysis in which two minimum and maximum scenarios were proposed concerning the result obtained and by validation using the receiver operating characteristic (ROC) curve method. The areas with high susceptibility to geo-hazards triggers are located in the southeastern part of the region, in the proximity of the hydrographic network. Flood risk is the hazard with the highest recurrence. Localities with high suitability for spatial development have a central-western distribution. According to the applied model, sites for each locality included in the study area can be identified. The high and very high suitability classes account for 54% of the total area, while the unsuitable classes represent 15% of the area. However, the vulnerability of the area and the need for the study are generated by 35% of the settlements being located in areas with high susceptibility.

## 1 Introduction

Natural hazards are complex phenomena that can occur independently, simultaneously, or as cascading events in a specific region and are augmented by climate change, population growth, rapid territorial development, and thus increased community exposure [1,2]. Natural disasters have a significant impact on inhabited areas around the world. Developing countries are the most affected due to poor infrastructure and reduced capacity to manage crises with economic and social consequences [3].

The term “multi-hazard” was first introduced in Agenda 21 for Sustainable Development in International Policy, which states the relevance of “comprehensive multi-hazard research” in human settlement planning and management of exposed areas [4]. The concept was further used in the Johannesburg Plan and the Hyogo Framework for Action. It is based on a spatial approach to analyze relevant hazards to a specific territory [5].

There are two distinct approaches in the analysis of natural hazards: independent multi-hazard analysis [6] and multiple layering to identify multi-hazard exposed areas [[7], [8], [9]]. The scientific approaches employed in natural hazard analysis can vary based on the scale of analysis, ranging from global to local. These approaches involve identifying and defining relevant hazards within the specific context of the area and the objectives of the study [10,11].

Most studies have concentrated on the detailed analysis of a single potentially destructive natural phenomenon: floods [[12], [13], [14]], landslides [15,16], and earthquakes [17,18]. However, a region is affected not only by a specific natural hazard but also by a series of geo-hazards.

Multi-hazard studies are based on the Multi-Criteria Decision Making (MCDM) technique, which combines information from multiple levels to result in a single evaluation index [19]. To conduct a multidimensional analysis, it is necessary to employ a scientific methodology that enables the integrationof a large number of indicators that serve as conditioning factors or alternatives [20] to generate an evaluation index. Therefore, the method based on using a Geographic Information System (GIS) by integrating MCDM techniques allows combining geographic data with value judgments to obtain information for decision-making [21]. In MCDM analysis, one of the most widely used methods in multi-criteria spatial analysis is the Analytical Hierarchy Process (AHP) introduced and developed by Saaty (1977) [22]. The method has been applied and modified for various domains, being increasingly developed in the natural hazards evaluation [[23], [24], [25]]. This type of analysis is based on integrating the weights obtained in the GIS environment to develop methodologies with results that stakeholders can use. This indicator-based approach is subject to uncertainty. Its elimination is increasingly recognized due to the uncertainty in data, models, and expert judgments [26] and inherent natural variability [27]. To ensure results stability and minimize uncertainty, some adjustments to specific parameters are applied during the evaluation and distribution of results [28].

In Central European countries, Romania is one of the most affected by natural hazards (floods, landslides, and earthquakes) with a significant economic and social impact [29]. Recent decades have highlighted human communities' vulnerability through increased material damage and reduced capacity to absorb risk phenomena [30], especially in the eastern part of Romania, where significant changes in land use have been registered, leading to increased vulnerability [31].

This research was performed in the Moldova River catchment. Its versatility in relief conditions and territorial suitability has led to the concentration of communities in areas exposed to natural hazards. Over time, extreme events have been recorded within the basin [32]. Non-compliance with planning rules, lack of coercive measures, rapid territorial expansion, and insufficient infrastructure in terms of hydro-technical arrangements are the leading causes of increased damage in the study area [33].

The aim of the paper is to estimate the suitable areas for spatial development in relation to the natural geo-hazards occurring in the study area (floods, landslides, and earthquakes). Associated to this aim, the paper has the following objectives: i) identification of causal factors and their standardization; ii) application of the AHP method for each hazard and obtaining susceptibility maps; iii) obtaining the multi-hazard map and estimation of areas suitable for territorial development; iv) validation of the results using sensitivity analysis and ROC curve method.

The novelty of the study results from applying the analysis to a hydrologically undeveloped watershed, representative of the eastern part of the Eastern Carpathians, affected by many natural hazards. The multi-hazard analysis of a medium-sized catchment in an area with low data availability is a novelty in Romania.

The results will allow modeling and identifying suitable areas for territorial expansion with reduced risk. The utilization of cartographic materials, statistical data, methodologies, and specialized programs will be essential in obtaining the final results. These outcomes will serve as a prerequisite for elaborating valuable materials for development strategies in the Northeast Region of Romania.

## 2 Materials and method

### 2.1 Study area

**Fig. 1:**
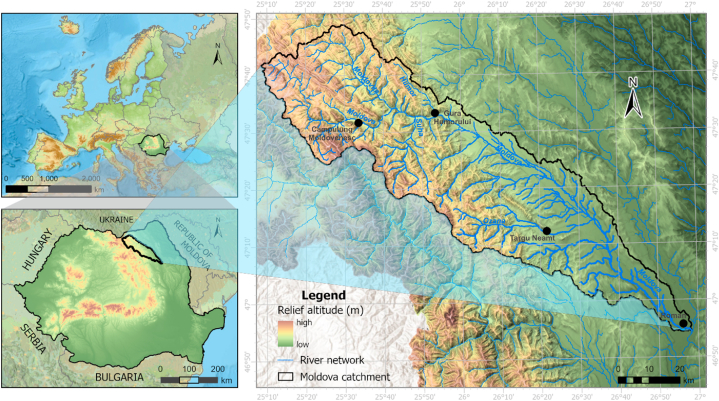
Geographical location of the study area.

The Moldova River basin is located in the eastern part of Romania (Fig. 1). The drained area of more than 4300 km^2^ extends over a wide altitudinal range between 180 m (in the confluence area with the Siret River, in the east) and 1858 m (maximum altitude, in the west), together with the specific geological conditions of the mountainous and submontane areas developed in the flysch zone and crystalline rocks. Climatic conditions generate a hydrological regime with high values of river runoff [34]. During the spring, the values amount to more than 45–50 m^3^/s (due to the melting of the snow cover in the mountain area) and with a minimum reaching 10–15 m^3^/s (due to the confinement of precipitation in the form of snow cover and ice) during the winter [35]. Climatic conditions with average annual temperatures of 9 °C in the lower basin and 2 °C in the upper basin and average rainfall ranging from 1200 mm in the high mountain area to 650 mm in the confluence area [36], together with the extreme characteristic of a temperate continental climate with heavy rainfall in short intervals during summer [37], generate frequent floods and flash-floods (more than 650 m^3^/s in the upper basin and over 1400 m^3^/s in the lower basin). These extreme events affect the main stream valley and tributary valleys and generate landslides, which have an extensive distribution within the catchment.

The study area is characterized as a transition zone between the mountain and the plateau, where the Subcarpathians make this transition. The Subcarpathians represent the young and dynamic relief units (the most recent orogeny of the Carpathian) with frequent landslides [38] and against deforestation after 1990. The plains in this area have provided a favorable natural setting for the development of human settlements (97 territorial-administrative units), reflected in the high density of population and settlements from the study area [39].

The changing economic, social, and political system in Romania has led to the need for a significant change in land use planning. The main problems identified were the need for a strategic vision, the lack of clear objectives, and the need for an implementation plan. At the national level, the Territorial Development Strategy of Romania is the document that provides the basis for the entire spatial planning system, supporting strategic documents at the regional, county, and local levels and ensuring their coherence.

The North-East Regional Development Plan 2021–2027 has several priorities, including a specific objective related to the development of climate change adaptation and risk prevention, where many projects are mentioned that aim to reduce the effects and damage to the population. The study area is predominantly rural, and the most effective measures are adopted within the Local Action Groups, which have been established in Romania since 2010. These public-private partnership structures aim to develop the area based on local development strategies.

The methodological framework includes the application of modern analysis methods and techniques that integrate GIS techniques, algebraic operations applied in GIS environment, spatial analysis, statistical data analysis, multi-criteria analysis, field observations, and focus groups.

### 2.2 Data used

**Fig. 2:**
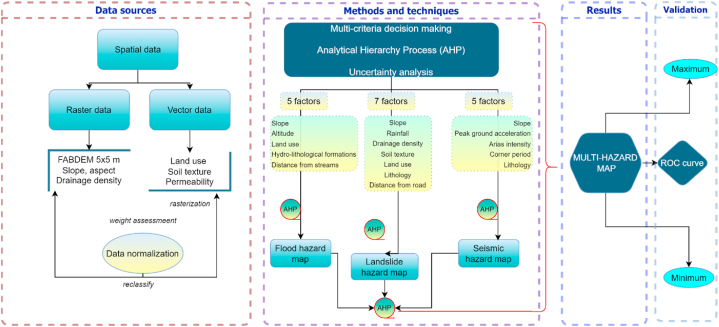
Methodological framework and workflow.

The favorability of the land for spatial development was estimated using the numerical terrain model obtained from the FABDEM (Forest and Buildings removed Copernicus DEM) database. This estimation was conducted on a global scale with a pixel size of 30 × 30 m, excluding forests and clusters. The superiority of this model, compared to other existing global DEMs, is attributed to the implemented corrections [40]. From this layer, several parameters such as slope, elevation, slope orientations, aspect, and drainage density were generated (Fig. 2). Thematic layers on lithology, rock permeability, and soil texture were made by digitizing geological and pedological maps at 1:200 000 scale from the Institute of Pedological and Agrochemical Research, Bucharest (1990) [41], and the Geological Institute [42]. Land use was extracted from the Copernicus database, from the Corine Land Cover set, for 2018 [43] (the most recent dataset available). The precipitation distribution for the present study was generated from the ROCADA (Romanian Climate Dataset) dataset, which has national coverage with daily data for 53 years [44,45] and from ERA 5 model produced by European Centre for Medium-Range Weather Forecasts, within the Copernicus Climate Change Service (C3S) [46] for the 2014–2018 period. The seismic hazard analysis was carried out using the database provided by the Seismic Risk Assessment and Research Centre and the European Facilities for Earthquake Hazard and Risk (EFEHR) platform, which is part of the SHARE project [47].

In the areas with a significant risk for natural hazards occurrence, the materials are supplemented with data from orthophotoplans, satellite images, the limits of the administrative-territorial units, and data acquired from the INS website – Tempo-Online at the local administrative units level. GPS data was collected from the field to delimit certain exposed areas and apply the focus groups for hazard analysis. The database on landslides and floods was collected during the fieldwork from specialized institutions or bibliographic resources that have studied these phenomena in the studied area.

### 2.3 Multi-criteria analysis – AHP method

The Analytic Hierarchy Process (AHP) was used to evaluate the susceptibility of areas to geo-hazards and their suitability for spatial development with regard to the occurrence of natural disasters. This method represents a multi-criteria approach in which pairwise comparison matrices and eigenvector analysis are used to establish the weights of the integrated factors within the problem addressed.

The method is based on a linear correlation of the selected factors. The first step involves a pair-wise comparison matrix where the inputs reflect the relative importance of the factor compared to the other factors analyzed. The pairwise comparison of the alternatives is performed in a matrix model, based on a reference scale proposed by Saaty (1977) [22], with values from 1 to 9, where the numerical values correspond to levels of importance (1 = equal; 3 = moderately; 5 = strongly; 7 = very strongly, 9 = extremely; 2, 4, 6, 8 = intermediate values). After the pairwise comparison step, the values are normalized, and the weights for each integrated factor are calculated [48].

The obtained results are validated by calculating the Consistency Ratio (CR) as an indicator of the degree of consistency or inconsistency, which quantifies the lack of ambiguity of the obtained weights (Eq. (1)) [49]:

(1) C.R.=CI/RI

where: C.I. – Consistency Index; R.I. – Random Index, where the value depends on the number of factors included in the matrix. The following formula is used to calculate the Consistency Index (C·I.) (Eq. (2)):

(2) C.I.=(λ_max−n)/(n−1)

where: *λ*_max_ – is the largest eigenvalue of the matrix; n – matrix size.

The consistency ratio is calculated to verify the weights obtained and avoid discrepancies within the matrices. According to Saaty (1977) [22], when the CR is less than 0.1, the weights are appropriate; otherwise, the matrix is re-evaluated [50].

The relevance of each thematic layer in relation to the other identified layers on the assessment of each hazard was provided by assigning weights. This was done using the relevant literature, observations made during the field phases, and expert judgments to complete the related matrices.

In order to obtain the result of the multi-hazard analysis for the Moldova river catchment, three matrices were made in the first phase for each hazard (floods, landslides, and earthquakes), followed by a comparative analysis of the results obtained to estimate the areas with optimal suitability for territorial sprawl. Based on the result (multi-hazard map), a sensitivity analysis was carried out in which spatial variations in development areas were tracked, considering the related degree of uncertainty.

The spatial database was created using ArcGIS Pro 3.0.3 software (ESRI 2022), and all parameters, were standardized and converted from vector to raster at a spatial resolution of 30 × 30 m per pixel.

### 2.4 Multi-hazard inventories and causative factors

The study can be analyzed from two perspectives. The initial section focuses on the analysis of natural hazards and the identification of areas with high susceptibility. In the second part, the analysis aims at identifying suitable areas for spatial development using multi-hazard mapping and sensitivity analysis.

In order to develop a model to assess and identify areas exposed to natural hazards with the potential to occur, a set of conditioning factors must be identified [51].

According to the current literature and the environmental characteristics of the study region, 13 causal factors were identified. The first step is to classify all factors by standardizing them to a uniform scale for comparison. The standardization used in the study is based on integers from 1 to 5 [52]. Therefore, value 1 represents the most stable condition and value five was given to the classes with the highest hazard susceptibility. This approach is quantitatively relevant and accurate when applied to causal factors [53]. Each factor was divided into different classes with specified threshold values, and their determination was based on field observations, mapping materials, and literature [54,55].

### 2.5 Flood hazard assessment map

The analysis is based on the fact that more than 88 extreme hydrological events have been identified in the Moldova river catchment, affecting communities from 1950 until now. For many of these events, data on the flooded area are available, but not other hydraulic parameters such as runoff velocity and water depth; therefore, it was necessary to develop the flood hazard map by applying the indicator-based AHP.

**Fig. 3:**
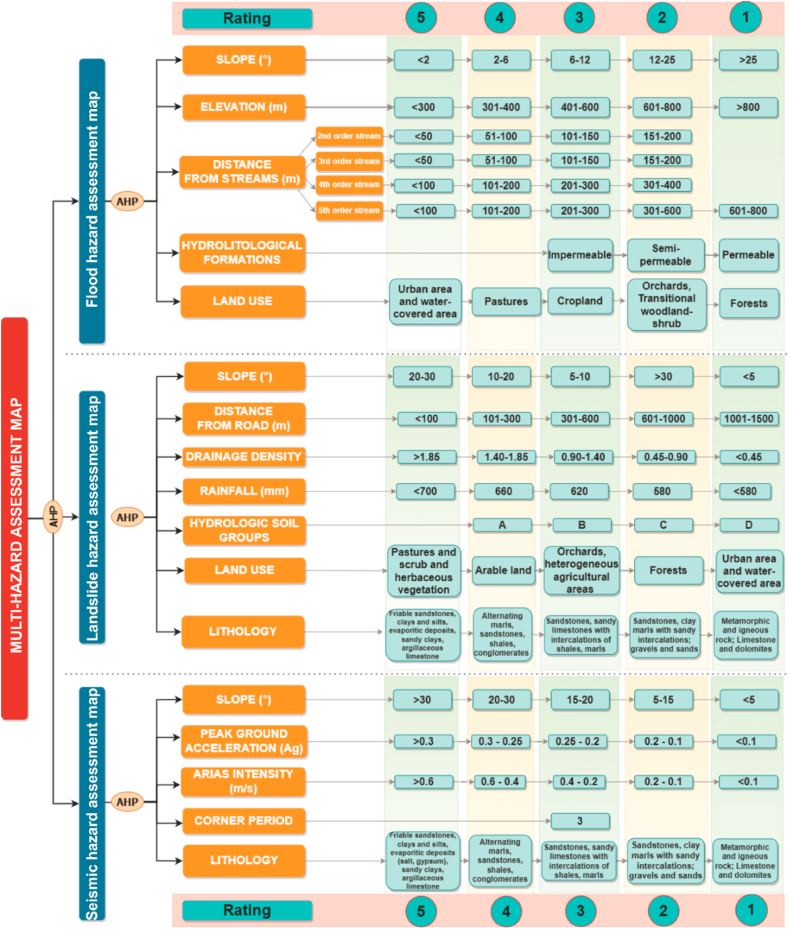
Weight assessment.

**Fig. 4:**
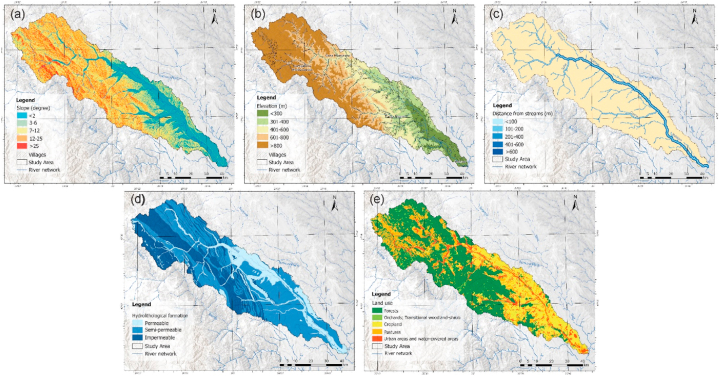
Thematic layers of the causal factors that contribute to flood hazard map (a. slope, b. elevation, c. distance from streams, d. hydro-lithological formations, e. land use).

A multi-criteria model based on the following control factors was applied to produce the flood hazard assessment map:

**Slope (Sl).** Slope distribution significantly impacts the evaluation of floods, flash-floods and associated landslide occurrence [56]. Areas with low slopes are susceptible to flooding. The related classes are located in the southeastern part of the study area near the main river course and tributaries (Fig. 4a), areas affected by flooding. Slopes were classified into five classes. Areas with low slopes favoring water accumulation were given the highest rating, and those with slopes greater than 25° were located at the opposite pole (Fig. 3).

**Altitude (Al).** Altitude can be considered one of the most relevant factors, together with slope, because flat areas have a high potential for flooding due to the amplification factor [57] and control the direction of water runoff [58]. Considering the spatial distribution of elevations within the basin, the elevation class below 300 m a.s.l. was assigned the maximum factor rating showing distribution in the southeast region, and the class with elevations above 800 m a.s.l. was assigned the maximum value (Fig. 4b).

**Proximity to river (Pr)** is a primary factor in analyses of the distribution of flood hazard, with the most affected areas being close to river courses as a consequence of flood reversal. The drainage network was generated based on the DEM used in the analysis. The watercourses were classified using the Strahler (1957) method, where the main channel is of order V. Order I streams were considered to have a low contribution to flooding and higher orders (II–V) were included in the analysis. Thus, for orders II and III, buffer zones were made at 50 m, 100 m, 150 m, and 200 m; for orders IV and V, buffers were made at 100 m, 200 m, 300 m, 600 m, and 800 m (Fig. 3). The rating factor was given according to the river's proximity. Areas in the river's proximity were given the maximum rating (Fig. 4c).

**Hydro-lithological formations (HLF).** The geological formations in the study area have been classified according to the hydrogeological behavior of the rocks they are composed of: permeable formations (e.g. sandstones, loess deposits, gravels, and sands) with a significant distribution near riverbeds, semipermeable formations (e.g. sandstones, loess deposits, gravels, and sands) with a significant distribution near riverbeds, semipermeable formations (e.g. sandstones, loess deposits, gravels, and sands) with a significant distribution near riverbeds: clays, breccias, sandstone within flysch-type deposits), impermeable formations with a distribution in the north-western part of the studied area (where basalts, andesites, limestones - flysch-type shale, crystalline limestones predominate) (Fig. 4d) [59]. The impermeable rock category received the highest rate because it affects the infiltration rate and supports the occurrence of floods.

**Land use (LU).** Land use is essential to flood occurrence due to the negative correlation between vegetation density and rainfall [60]. The spatial distribution of land use was derived from the Corine Land Cover dataset for the year 2018 and classified according to the influence of flood hazard distribution. Land use and land cover play an important role in rainfall interception, infiltration, evapotranspiration, and groundwater retention capacity in certain areas [61,62]. In this context, built-up areas received the highest score due to high impermeability and water-covered areas, followed by grassland, cultivated land, and orchards. The forest category received the minimum score rating (Figs. 3 and 4e).

Following the reclassification of thematic layers, the AHP pairwise comparison matrix (Table 1) was utilized to conduct an assessment of flood hazards. A cross-correlation was performed in a 5 × 5 matrix to obtain the weights for each factor. The values obtained were validated by the Consistency Ratio (C.R.) and shown by the obtained value of 0.08 that the judgments were well evaluated [63].

**Table 1:** The weighting coefficient for every factor and the Consistency Ratio (CR) for flood hazard assessment.

	F1	F2	F3	F4	F5	Weights
**F1**	1	4	1/2	3	3	**0.281**
**F2**		1	1/3	1/2	1/4	**0.069**
**F3**			1	3	3	**0.372**
**F4**				1	1/3	**0.098**
**F5**					1	**0.181**

CR = 0.08; <0.1

where: F1 – slope, F2 – altitude, F3 – proximity to river, F4 – hydro-lihological formations, F5 – land use.

After obtaining the weights of the factors included, the map of the *flood hazard index* (FHI) is calculated using the following equation (Eq. (3)):

(3) FHI=0.281*Sl+0.069*Al+0.372*Pr+0.098*HLF+0.181*LU

### 2.6 Landslide hazard assessment map

Landslides are triggered in areas where several causal factors combine, particularly in mountainous and hilly areas and densely populated areas, and are one of the recurrent natural problems that are widespread worldwide and have caused significant damage [64]. Thus, human-induced pressure and territorial expansion directions can increase landslide occurrence risk.

Landslide analysis has two main approaches regarding the type of analysis and triggers: i) local scale studies based on investigation and inventory; ii) regional analyses that consider a range of geological, geomorphological, climatic, hydrological, and land cover factors [65].

Factors considered in the susceptibility analysis of landslides are the terrain slope, which is one of the most important causal factors of landslide processes, lithology due to the alternation of permeable and impermeable types of rocks, vegetation cover that can affect soil humidity or pressure from anthropogenic factors, rainfall, drainage density and distance from the national road network [66].

The study area is located between the mountain and the plateau, and the transition is made through the Subcarpathians. The Subcarpathians are the most representative of landslides in Romania due to their typological complexity as a strongly tectonised pre-Miocene-Miocene subunit (characterized by asymmetric and fractured faults, often forming monoclinal surfaces) [67].

**Fig. 5:**
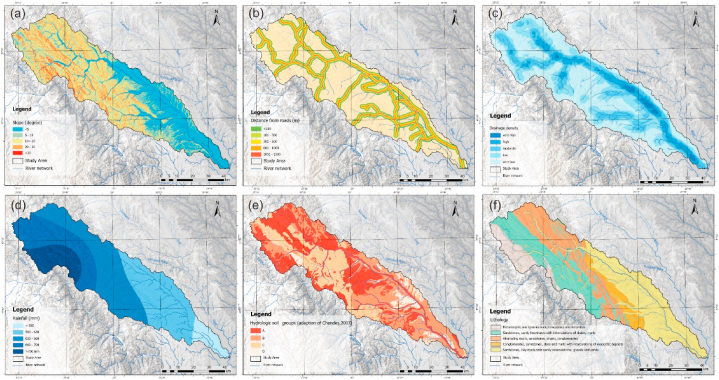
Thematic layers of the causal factors contributing to landslide hazard map (a. slope, b. distance from roads, c. drainage density, d. rainfall, e. hydrologic soil groups.

From this point of view, the occurrence and spatial evolution of landslides are related to several trigger factors:

**Slope (Sl).** The slope increase leads to their occurrence, being inversely proportional to the flood situation. Thus, the gravity force related to shear stresses increases, and susceptibility increases with slope classes [68]. In the present study, landslide movements were considered, excluding other landslides (such as rock falls). In these considerations, slopes between 20 and 30° are considered the most susceptible in the development of landslides [69] and received the maximum score rating (Figs. 3 and 5a), slope classes with values lower than 5° being on the opposite side in achieving standardization of this factor.

**Distance from road (Dr).** Depending on local conditions, road modifications are a critical intervention that can lead to landslide triggering and extension. Territorial expansion leads to an increase in the density of the road network and several slopes in dynamic equilibrium being transformed into landslide-ready areas. The road network was digitized on 1:25 000 topographic maps and updated using orthophotos. Consequently, buffer zones were established around the road network [70]. The areas near the roads being considered the most prone received the highest score (class 0–100 m), and with distance from them, the susceptibility decreased.

**Drainage density (Dd).** High drainage density decreases water infiltration and causes more rapid surface runoff. At the same time, high rainfall followed by soil saturation on a steep slope leads to landslide triggering/reactivation [71]. Thus, areas with high drainage network density received the highest rating due to the higher probability of landslide triggering (Fig. 5c).

Rainfall (Rf). is one of the essential factors in landslide occurrence, and slope response/instability varies depending on lithological, pedological and vegetation cover characteristics [72]. The Inverse Distance Weight (IDW) method was used to generate the distribution of mean annual rainfall and is considered the most appropriate method for rainfall interpolation [73]. The result obtained was classified into five classes, and the class with rainfall above 700 mm received the highest value (Fig. 5d).

Hydrologic soil groups (HSG) or soil texture is the relative proportion of sand, loam and clay content that has a major impact on how landslides form. Soil types were classified into four groups (A - D) and adapted to the Romanian system [74]. The highest value (4) was attributed to soil type with sandy, loamy and derived variants, in opposition to the soil classes, which are characterized by a loamy-clayey texture, which received a rating of 1 (Fig. 5e). Soils with a high clay content form stable and resistant to detachment aggregates. On the other hand, light soils such as sandy soils or coarse clays are easy to loosen because they have a low organic matter content, which leads to their inability to form stable aggregates. Thus, soils with higher sand content, steep slopes, and intense rainfall are favorable conditions for landslides [75].

**Land use (LU)** is an important factor in landslide susceptibility and directly or indirectly affects some processes, such as surface runoff, evaporation or infiltration [76]. Slope instability is directly related to land use. Pastures, land covered with rare tree associations, and poorly managed orchards provided weak slope stability [77] and received the maximum value (Fig. 3). At the opposite pole are built-up areas and forest-covered areas, which have low susceptibility to landslides [78]. However, anthropogenic activities such as deforestation, excavation of upper slopes, land use change, lack of soil erosion and slope stabilization measures lead to landslide triggering.

**Lithology (Lt).** The main criteria considered for the evaluation of lithological classes were the genesis of geological formations and their geotechnical properties. The geological factor significantly controls landslide distribution (GT 019–98 [79] presented in Refs. [80,81]). The basin surface developed on molasse deposits is the most affected by landslides. These deposits belong to the Subcarpathian Nappe formed by friable sandstones, clays and silts, evaporite deposits (salt, gypsum), sandy clays, argillaceous siltstones [82] received the highest rating (Fig. 3). Score 4 was given to the marl-sandstone flysch composed of alternating marls, clays and sandstones. The sandstone flysch, wildflysh, received a rating of 3, the Volhinian and Basarabian deposits of the Moldavian Plateau and the Quaternary riverbed deposits received a rating score of 2. The most compact and stable are the metamorphic and magmatic rocks of the Crystalline-Mesozoic zone of the Eastern Carpathians, which is why this area was assigned a rating score of 1.

Considering the conditioning factors analyzed, the AHP method was applied, and a pairwise comparison matrix (Table 2) was performed to determine the weights and check the judgments by calculating the CR.

**Table 2:** The weighting coefficient for every factor and the consistency ratio (CR) for landslide hazard assessment.

	F1	F2	F3	F4	F5	F6	F7	Weights
**F1**	1	4	1/3	5	4	4	4	**0.255**
**F2**		1	1/3	1/2	1/4	1/3	1/4	**0.058**
**F3**			1	4	5	3	4	**0.320**
**F4**				1	4	3	2	**0.128**
**F5**					1	1/3	1/3	**0.039**
**F6**						1	1/2	**0.089**
**F7**							1	**0.111**

CR = 0.09; <0.1

where: F1 – lithology, F2 – drainage density, F3 – slope, F4 – rainfall, F5 – hydrologic soil groups, F6 – distance from road, F7 – land use.

After obtaining the weights of the factors included, the map of *landslide hazard index* (LHI) is calculated using the following equation (Eq. (4)):

(4) LHI=0.255*Lt+0.058*Dd+0.320*Sl+0.128*Rf+0.039*HSG+0.089*Dr+0.111*LU

### 2.7 Seismic hazard assessment map (Geotechnical vulnerability)

In seismically active regions, landslides can occasionally occur as a result of earthquakes, leading to significant landslides either during or after the seismic event. Due to the proximity of the Vrancea seismic zone, shocks induced by strong earthquakes have been an important trigger for such events over time [77,83].

The Vrancea seismic zone is responsible for 90% of the seismic activity in Romania, releasing over 95% of the total seismic energy [84].

For the analysis of earthquake hazards, the most pertinent factors were selected based on the study's applicability and previous analyses. These factors include slope, peak ground acceleration, corner control, arias intensity, and lithology. **Peak ground acceleration** (PGA). Ground motion intensity characteristics can be measured by physical parameters such as PGA. A series of empirical equations have been proposed to quantify seismic resistance at design time to correlate intensity with PGA.

**Fig. 6:**
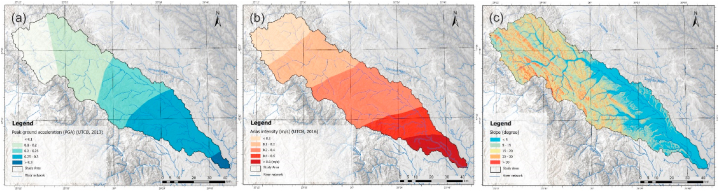
Thematic layers of the causal factors that contribute to seismic hazard map (a. peak ground acceleration, b. arias intensity, c. slope).

The PGA is the maximum absolute value recorded from a given location [85]. Seismic hazard is described by the peak value of the horizontal seismic acceleration of the ground ag determined for an average recurrence interval [86]. The seismic zonation map of the P100-1/2013 standard on the Romanian territory regarding peak ground acceleration values for ag design with an average recurrence interval of 225 years and 20% probability of exceedance in 50 years with values between 0.3 and 0.1 a_g_ was considered for the studied area. Five classes were separated, where the class higher than 0.30 a_g_ received the maximum rating, being located in the south-eastern limit of the study area, with the opposite side being the class with ag values lower than 0.1 (Fig. 6a).

**Arias intensity (Ai)** was based on data presented in studies conducted by UTCB (2016) [87]. Seismic recordings of the parameters from earthquakes in the Vrancea seismic zone on 4 March 1977 (Mw = 7.4 and h = 94 km), 30 August 1986 (Mw = 7.1 and h = 131 km), 30 May 1990 (Mw = 6.9 and h = 91 km) and 31 May 1990 (Mw = 6.4 and h = 87 km) were used to determine this parameter. For the analyzed area, the parameter Ai shows values of 0.6–0.1 m/s (Fig. 6b).

**Corner control (Tc)** of the response spectrum represents the boundary between the area (range) of maximum values in the relative velocity spectrum and the area (range) of maximum values in the relative displacement spectrum [86]. The whole area studied overlaps over Tc = 0.7 s; at the Romanian level, the maximum is 1.6 s.

**Slope (Sl)** is an important factor in earthquake hazard, especially in slope instability, and is closely related to landslide occurrence. In this context, slopes with values greater than 30° have been assigned the highest class and slopes with values less than 5° are the lowest.

**Lithology (Lt).** The classification used in that natural hazard is similar to that used in the landslide analysis due to the fact that the geotechnical properties of the rocks were taken into account in the assessment.

In the seismic hazard scenario, a 5 × 5 matrix was performed where cross-comparison between the identified factors was performed to obtain the corresponding weights (Table 3). The values obtained were verified by calculating the Consistency ratio indicating the consistency of the judgments.

**Table 3:** The weighting coefficient for every factor and the consistency ratio (CR) for seismic hazard assessment.

	F1	F2	F3	F4	F5	Weights
**F1**	1	1	3	3	3	**0.326**
**F2**		1	2	4	3	**0.318**
**F3**			1	3	1/3	**0.137**
**F4**				1	1	**0.087**
**F5**					1	**0.133**

CR = 0.05; <0.1

where: F1 – Arias intensity, F2 – Peak ground intensity, F3 – slope, F4 – Corner control, F5 – Lithology.

The weights obtained from the application of multi-criteria analysis were integrated into the GIS database for the generation of the seismic hazard map (SHI) using the following equation (Eq. (5)):

(5) SHI=0.326*Ai+0.318*PGA+0.137*Sl+0.087*Tc+0.133*Lt

### 2.8 Multi-hazard map and sensitivity analysis

The survey is essential due to the possibility of multiple natural hazards occurring simultaneously or cascading within an area, resulting in significant impacts on communities. Increasing the resilience of these communities can be achieved through planned territorial expansion that considers these disasters that may occur. Since, depending on the analyzed area, geo-hazards can occur with different intensities and magnitudes [58], through the AHP, the weights related to the obtained results were established. Since numerous hydrological events were recorded in the study catchment, this hazard received the highest weighting (Table 4). The second value is attributed to landslides, which show a median distribution within the basin due to the generating conditions (lithology that favours the process being a tectonic transition zone, slopes, and land use). Associated with these events, it is also considered necessary to analyze the seismic hazard; their frequency and intensity in the studied area are relatively low. However, earthquakes in the 1940s and 1990s were felt in the southern part of the area.

**Table 4:** Weights of natural hazards included in the analysis and the change of their values (ΔW_i_).

	F1	F2	F3	Weights	ΔW_i_
**F1**	1	1/4	1/5	**0.098**	**0.0196**
**F2**		1	1/2	**0.334**	**0.0667**
**F3**			1	**0.568**	**0.1135**

CR = 0.02; <0.1

where: F1 – seismic hazard; F2 – landslide hazard; F3 – flood hazard.

Thus, in order to spatialize and estimate areas suitable for territorial sprawl, the results obtained by applying the methodology described above were integrated into the GIS environment using the weighted linear combination method (Eq. (6)):

(6) MHM=0.098*SHI+0.334*LSI+0.568*FHI

Sensitivity analysis looks at the variation in uncertainty in the model output due to variations in the input parameters. The sensitivity analysis of the output is included for a comprehensive planning process evaluation. Each input factor included in the analysis is subject to uncertainty that may affect the proposed model output [88]. In order to take these uncertainties into account, a series of predictions can be made. In this case, two scenarios on the range of weight deviations were made by altering the weights by 20% (addition and subtraction) [19] compared to the initial weight of the factors (Table 4).

Considering the error ΔS, which is a consequence of the independent errors ΔWi, the weight coefficient is computed by the following equation (Eq. (7)) [23,89]:

(7) $$\Delta S=\sqrt{\sum_{i=1}^{n}{\left({\Delta W}_{i}X_{i}\right)}^{2}}$$

After calculating the ΔS error, the raster file obtained was multiplied by 1.96 to achieve a 95% confidence interval [8]. The result is added to and reduced from the base file (area estimates on spatial expansion). Thus, two raster files were generated with two extreme scenarios representing the minimum and maximum relative to the initial result.

Uncertainties can be due to inherent natural variability and statistical uncertainties model, leading to different levels of natural hazard uncertainty. Integrating uncertainty into analyses is necessary for probabilistic risk assessment [27].

## 3 Results

**Fig. 7:**
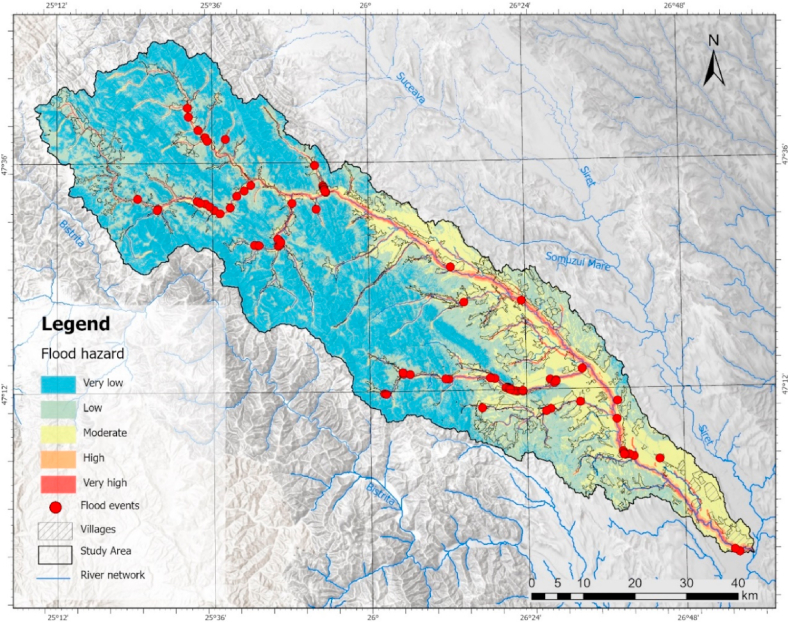
Flood hazard zonation map.

A GIS-based MCDM analysis generated a map of flood hazard spatialization within the Moldova catchment. Five flood hazard classes have been derived using the standard deviation as a classification method of the obtained file (Fig. 7). Based on the obtained results, it is evident that the areas in close proximity to rivers exhibit the highest vulnerability. Thus, the high and very high vulnerability class is associated with 8.26% of the total area of the basin and 23% of the area within the urban area of the localities since their longitudinal distribution of the hydrographic network (Table 5) indicates a major vulnerability, which is demonstrated by the hydrological events that have occurred in this area.

**Table 5:** Percentage per flood hazard class.

Flood hazard	% total	% intravilan
Very low	40.12	1.47
Low	31.74	28.54
Moderate	19.85	46.91
High	4.84	14.33
Very high	3.42	8.73

The moderate hazard class is predominant in distribution within the urban area, with a percentage of 47%. It is distributed from the contact with the mountain area, related to the Moldavia Subcarpathians and the Suceava Plateau.

**Fig. 8:**
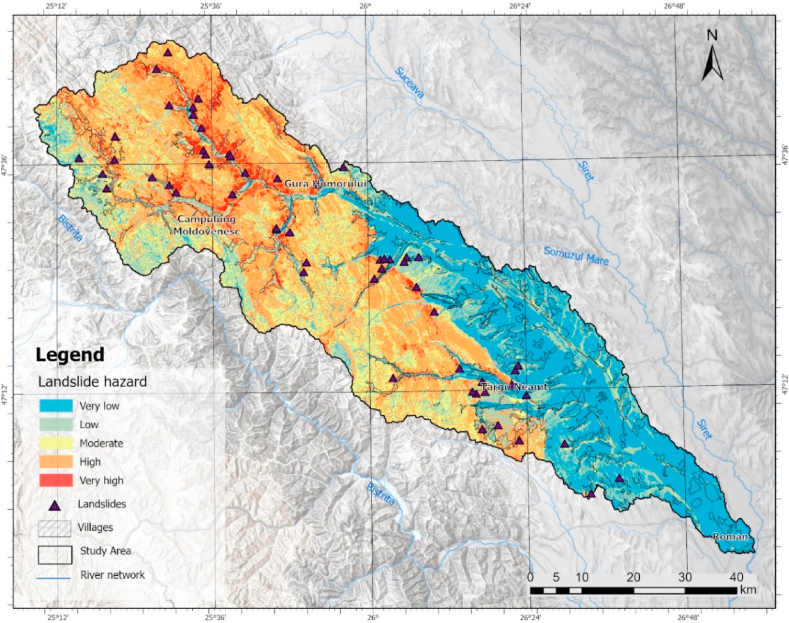
Landslide hazard zonation map.

The landslide susceptibility distribution was generated by integrating the weights obtained from the AHP methodology into the GIS environment. The same classification method was used, the standard deviation highlighting the oscillations from the mean value. The representation of five hazard classes allows the analysis of the hazard distribution (Fig. 8).

Hence, it is evident that the northeastern part of the area displays a greater susceptibility to landslide hazards, particularly on the hillsides near the rivers within the mountainous region, as well as in the contact area of the Subcarpathians. The highest degree of hazard covers 4.87% of the analyzed area and 3.1% of the locality area (Table 6). The highest landslide susceptibility value belongs to the high hazard class with a value of about 28% due to favorable relief conditions. However, in the area of the settlement it shows a value of 12%.

**Table 6:** Percentage per hazard class for landslides.

Landslide hazard	% total	% intravilan
Very low	28.10	52.66
Low	18.65	23.26
Moderate	19.42	8.74
High	28.97	12.21
Very high	4.87	3.10

**Fig. 9:**
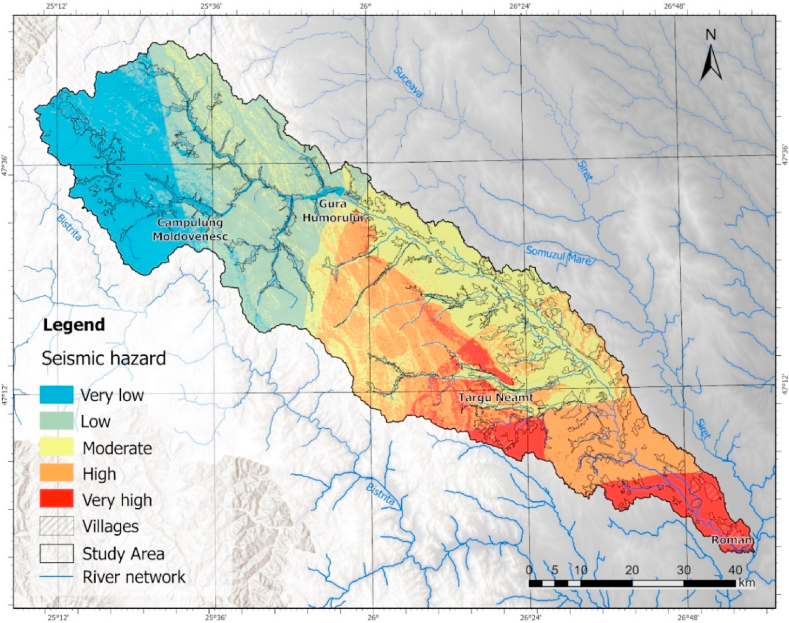
Seismic hazard zonation map.

In terms of seismic hazard analysis, upon implementing the methodology and categorizing the results, it becomes apparent that the areas situated in the southern part of the basin are most impacted owing to their proximity to the Vrancea seismic region. (Fig. 9). Furthermore, the susceptibility is the lowest in the north-western extremity of the catchment, which overlaps the mountain area.

**Fig. 10:**
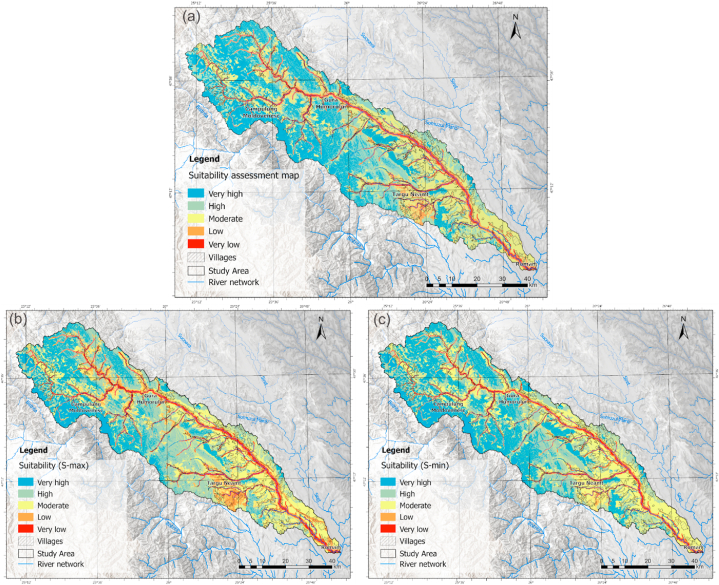
Land suitability distribution for spatial development (a) and scenarios for maximum (Smax) (b) and minimum (Smin) (c) values from sensitivity analysis.

The multi-hazard analysis carried out is a novelty in Romania and can be used as guidance on the territorial development directions of the localities in the study area. In addition to the result obtained from the direct application of the AHP methodology and the integration of the three geo-hazards, a calibration of the model was performed through sensitivity analysis which includes the consideration of the uncertainties that may occur in the assignment of the value, in the representation of the result (Fig. 10a). Thus, applying the method proposed by Bathrellos et al., 2017 [8], two scenarios illustrating the minimum and maximum values (Fig. 10 b,c), compared to the initial result and based on a 95% confidence interval, were performed.

The obtained map was classified using the same number of classes, and the analysis aims to identify the directions of territorial development concerning the three representations.

Regarding the distribution of areas suitable for territorial expansion, the northwestern part of the basin predominantly exhibits favorable classes, whereas the southeastern portion, specifically the lower course area, demonstrates low suitability. This is principally caused by floods near the confluence with the Siret River, Subcarpathians that have developed on the molasse deposits that provide instability, and the seismic hazard due to the proximity of the seismic epicenter on the Romanian territory.

A comparative analysis of the results shows that within the very high suitability class, there is a difference of 8% of the area analyzed between minimum and maximum suitability (Table 7). Within the intermediate classes, there are no significant changes in terms of distribution in the territory.

**Table 7:** Percentages representing the area of each suitability class.

Suitability zone	S(%)	S_max_ (%)	S_min_ (%)
**Very high**	20.53	17.82	25.23
**High**	33.59	33.87	32.47
**Moderate**	31.85	30.38	30.89
**Low**	7.96	11.17	6.23
**Very low**	6.07	6.77	5.18

The areas with low suitability zones class have a 5% difference between the minimum and maximum. They are controlled by geological structure, slope and the fact that these areas are located close to the river network, therefore flood hazard areas. According to Fig. 10, these differences are highlighted in the Ozana basin near Targu Neamt.

Furthermore, to validate the obtained results, a comprehensive inventory of hydrological events and landslides in the Moldova catchment was conducted. This inventory relied on field observations, satellite images, and relevant literature.

**Fig. 11:**
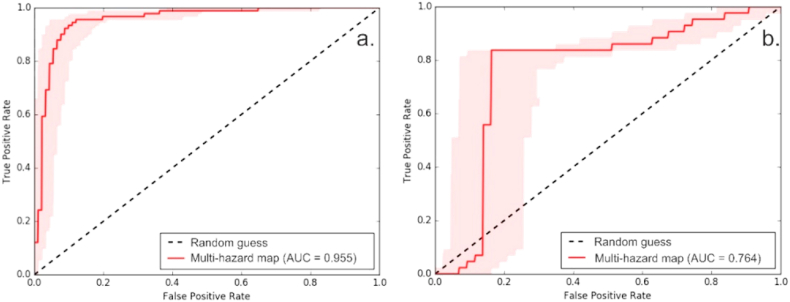
ROC curve for quantitative validation a. flood; b. landslide.

The receiver operating characteristic (ROC) curve method validated the multi-hazard map obtained. The ROC curve for the proposed model was performed to validate the model in terms of both flooding and landslides. ROC is a graphical representation of the true-positive and false-negative rates, considering all possible variants of existing flood and landslide data against the model prediction results [90]. The results of the ROC curve are shown in Fig. 11, where the area under the curve (AUC) was estimated to be 95.5% for floods and 76.4% for landslides, indicating the accuracy of the proposed model.

Through the application of this validation method, it can be concluded that the GIS-based MCDM application is a suitable model for estimating and assessing the susceptibility to natural hazards in areas where data availability is limited.

## 4 Discussions

The results derived from this study could be an essential contribution to land-use planning by integrating risk categories that generate particular problems for anthropized areas. The sprawl of these spaces in areas where natural hazards occur without adequate spatial planning implies the occurrence of events that affect local communities.

Romania's territorial development strategy, Polycentric Romania 2035 is the long-term programmatic document that defines the territorial development vision of the national territory for the time horizon 2035 and sets development objectives, measures, actions, and concrete projects at the territorial level.

The Ministry of Environment is the coordinator, and the County Councils are legally required to produce risk maps. Given the situation in recent years, the flood risk maps are among the most sensitive, and there has been an urgent need to produce them following legal obligations. All these measures also came as an obligation of the introduction of European directives (Directive 2007/60/EC).

For example, floods are one of the most destructive natural hazards at the European level. Despite early warning systems developed in recent decades, the damage has increased substantially as flood-vulnerable areas have been included in urbanized spaces [91]. The highest number of floods on the Romanian territory have occurred in the last three decades (1990–2020), with a maximum recorded in 2005–2010 [92,93]. The analysis of significant floods highlights the occurrence of several extreme hydrological events in recent years in order to respond directly to global climate change [94] and anthropogenic impacts [95,96] generating record high water levels [97]. During this period, exceptional hydrological events were also recorded in the Moldova catchment, culminating in the 2005 and 2008 floods that affected more than 20 communities. Under peak climate scenarios (e.g. a 4 °C increase in global average temperature by the end of the 21st century), climate change could increase direct flood damages threefold [98] unless concrete control and mitigation measures are taken. A spatial assessment of this risk is thus required in line with the need to implement local warning systems for the population and to restrict urban expansion in vulnerable areas.

Following historical floods in the Moldova river basin, landslides were reactivated in significant areas. The phenomenon is specific to the transition areas from the Carpathian to the plateau in the eastern part of the Carpathians, being generated by the geological structure favorable to the production of these processes and by the reactivation of some areas due to the increase in rainfall intensity generated by regional climate change [99]. The landslide density in the Moldova catchment ranges from 0 to 10 landslides per 100 km^2^ in the lower basin to 51–120 landslides per 100 km^2^ in the upper basin [67]. Most of these landslides do not affect human communities with high population densities. However, several areas are vulnerable to landslides in the mountainous area of the basin (upper basin of the Ozana River, Moldovita River basin or upper basin of the Moldova River). The hazard analysis and the vulnerability to landslides should also be carried out based on future climate scenarios, as the magnitude of the events is currently relatively low. However, an increase is not excluded due to climate change or extended anthropogenic impacts [100].

Different neotectonic uplift movements affect eastern Romania's Carpathian, sub-Carpathian, and neighboring areas [101]. These imprint a high seismic risk for the eastern part of the Carpathians, with medium-depth earthquakes mainly related to the Vrancea seismic source located in the SE Carpathian Curvature sector [102]. Due to its deep focal regime, the Vrancea seismic region is also responsible for the occurrence of landslides, processes distributed over a broad region [69].

This region also includes the Carpathians and Moldavia Subcarpathians and the central and southern sectors of the Moldavian Plateau. Following major earthquakes in 1940 (Mw7.7) and 1977 (Mw7.4), shallow landslides, rock falls, and rock slides have been triggered as co-seismic and post-seismic processes at distances of up to 100–200 km from the epicenter. These past earthquakes have generated significant landslides [65,103], such natural and future hazards that may affect human communities in this basin cannot be excluded.

The analysis of geo-hazards is an important issue when identifying spatial development directions. Thus, the methodology and methods used to reduce the degree of uncertainty through the two scenarios of minimum and maximum and the determination of the ROC curve validate the proposed model.

**Fig. 12:**
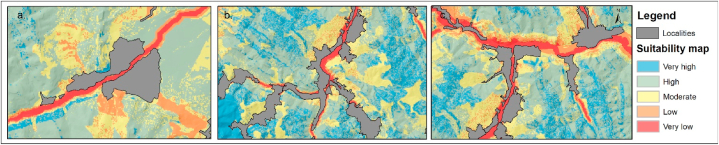
Suitability map – identification of territorial development directions for Slatina village (a), Stulpicani village (b) and Gura Humorului town (c).

Within the regional, county, or local spatial development plans, the types of risk are analyzed individually by presenting diagnostic elements and sub-objectives referring to some structural measures concerning the development of protective infrastructure, as well as non-structural measures referring to increasing the awareness of the population about the risks they are exposed to. Risk maps are required for urban and spatial planning studies, which will be used to determine the direction of development of settlements and the positioning of economic objectives. However, realizing individual maps by type of risk may produce inconsistencies when territorial development directions are proposed and require a larger number of maps with different hazard distribution information. Thus, synthesizing in a single map supports decision-makers by applying sustainable planning strategies [57]. Strategic directions for territorial expansion can be established based on the spatial distribution of natural hazards that may affect this area. Using the cartographic material, it can be seen that the appropriate direction for territorial expansion is predominantly east (Fig. 12a). In the second situation, the appropriate direction would be northwest (Fig. 12b) and predominantly southern for the third panel (Fig. 12c).

According to the statistical data obtained, 35% of the areas of the localities in this area are situated in areas with a high and very high susceptibility to natural hazards, and 42% are located in non-hazardous areas.

The map of the distribution of the suitability of territorial development can be a tool for institutions involved in planning and urban development (ministries, municipalities, prefectures, and research institutes).

The multi-hazard distribution map can be a valuable tool in early spatial planning as it provides a comprehensive view of the distribution of hazards associated with the area. The synthesis of hazards in a single material is a method that facilitates the decision-making process to implement sustainable strategies [104].

Geomorphological conditions, the hydrographic network, land use, and spatial accessibility are all factors that question the suitability for inhabitation within the area. However, climate change, land use change, deforestation, and uncontrolled building have increased the vulnerability of settlements and the necessity to carry out these studies.

The limitations of the study are that although the assessment of each parameter in the AHP methodology was carried out by standard agreement in terms of weight scores, a slightly different result can be obtained if specialists on a domain-by-domain analysis analyze them. Therefore, it can be recommended that all these natural hazard mappings be done in parallel by specialists from different fields, which would increase the reliability of the results. Another area for improvement is the need for complete spatial data to validate the results. Most of the localities in the catchment are located along the river network, which exposes them more to hydrological risks and less to landslide and earthquake risks.

The requirement for natural hazard studies arises from the need to mitigate impacts on communities. Uncontrolled spatial sprawl, chaotic distribution of buildings, lack of a coherent spatial development policy, lack of a substantiated and validated risk study makes it necessary to study these aspects for efficient risk management.

The political factor is the deciding factor. Promoting these results would make it easier for decision-makers to understand the issue's importance. Increasing resilience is vital in these types of risk situations, and thorough preparation at the government level to react accordingly is vital for both the population and institutions.

The consequences of climate change negatively impact communities, and regional sustainability and spatial development planning are directly affected by natural hazards. These changes raise complex issues and require approaches to interrelated factors that promote the development of a governance system that enables effective mitigation and planning strategies. There is discussion of decentralization, but steps to achieve it are slow. Decentralization would make governance work and improve resilience to crises. In other words, exactly what is needed to solve problems affecting communities. It is a political philosophy that can provide the basis for vital strategies and actions for local authorities. Governance would improve responsiveness and resilience to risks. After the abolition of collectivization, land use has suffered significant changes because the rigor of the communist regime, driven by appropriate agro-techniques, has been weakened. Against the backdrop of subsistence farming, it is difficult to control the proper use of land, so the emphasis should be on awareness, permissiveness, and not coercion.

The present study could serve as a basis for the current analyses on land expansion and the introduction of more specific variables on how to analyze in relation to area-specific geo-hazards. This approach can contribute to implementing an administrative-territorial reform initiated at a local or regional level, always postponed for various reasons [105]*.*

In Romania, the high population density in certain areas leads to an expansion of built-up areas near areas with a high degree of vulnerability, such as near riverbeds. Therefore, measures can be taken to limit land concessions and issue building permits in areas at high risk of natural hazards.

Possible strategies to reduce future vulnerability to this risk identify several application directions: increasing public awareness, establishing an early warning system and site analysis for future infrastructure and civil construction projects, including existing ones.

However, the study provides a reliable assessment of multiple hazards carried out individually and collectively for the Moldova catchment.

## 5 Conclusions

When discussing the territorial development of a specific region, this study has focused on two key aspects to be considered. The first is the spatialization of geo-hazards relevant to the studied area, namely for floods, landslides and earthquakes, based on the analysis of some conditioning/triggering factors. The second approach integrates the maps obtained for each natural hazard analyzed into a multi-hazard map.

Through the implementation of this approach, it becomes possible to generate results that can be incorporated into spatial planning documentation, taking into account the geo-hazards that communities may encounter. With the help of this cartographic material, it is possible to identify the suitable directions of territorial development for each settlement in the analyzed area, which is extremely useful in future spatial planning actions.

The GIS-based MCDM method implemented in the present study is based on the standardization and analysis of triggers and assigning weights to each factor by applying AHP. Since the criteria and the way of assigning weights can generate uncertainty, sensitivity analysis was applied by performing two scenarios.

The study results highlighted that the areas located in the southeastern part of the Moldova catchment are the most susceptible to natural geo-hazards being overlapped over the contact zone and the plateau (with low altitudes compared to the rest of the study area). The high vulnerability of the settlements is because most of them are distributed close to the hydrographic network, which provides a major risk of flooding, the transition zone of the Moldavia Subcarpathians being considered a dynamic area with frequent landslides. The southeastern end of the basin is the most prone to earthquakes (due to the direction of seismic wave propagation). In this context, 14% of the basin area is distributed in low and very low suitability classes, and 35% is situated within the settlement limits.

Areas suitable for land development are predominantly located in higher areas, with low and low natural hazards. Moreover, the application of this methodology enables the identification of areas with minimal exposure to natural hazards. Sensitivity analysis revealed minor differences in the spatial distribution of the two scenarios (minimum and maximum). The area with the most remarkable changes was located in the southwest of the basin and belonging to the Ozana river.

The obtained multi-hazard map was validated by correlating the results with the recorded events. The values of 0.95 for floods and 0.76 for landslides obtained from the ROC curves indicate that the proposed model is adequate.

Disaster risk management is an essential aspect of building resilient communities. The current study meets this objective by identifying areas that are suitable for territorial development, thereby reducing the impact of natural risks. At national level, this type of methodology meets the needs of the new National Recovery and Resilience Plan. At local level, the utilization of this type of tool, which integrates the geo-hazards specific to a particular area, assists the relevant authorities in identifying areas that are suitable for territorial development.

## Author contribution statement

Oana-Elena Chelariu: Conceived and designed the experiments; Performed the experiments; Analyzed and interpreted the data; Contributed reagents, materials, analysis tools or data; Wrote the paper.

Ionuț Minea: Conceived and designed the experiments; Analyzed and interpreted the data; Contributed reagents, materials, analysis tools or data; Wrote the paper.

Corneliu Iațu: Conceived and designed the experiments; Analyzed and interpreted the data; Wrote the paper.

## Funding statement

This work was supported by a grant of the Ministry of Research, Innovation and Digitization, 10.13039/100005186CNCS – 10.13039/501100006595UEFISCDI, project number PN–III–P1-1.1-PD-2019-0577, within PNCDI III.

## Data availability statement

Data will be made available on request.

## Additional information

No additional information is available for this study.

## Declaration of competing interest

The authors declare that they have no known competing financial interests or personal relationships that could have appeared to influence the work reported in this paper.
